# Minority stress, distress, and suicide attempts in three cohorts of sexual minority adults: A U.S. probability sample

**DOI:** 10.1371/journal.pone.0246827

**Published:** 2021-03-03

**Authors:** Ilan H. Meyer, Stephen T. Russell, Phillip L. Hammack, David M. Frost, Bianca D. M. Wilson

**Affiliations:** 1 The Williams Institute at the School of Law, University of California, Los Angeles, CA, United States of America; 2 Department of Human Development and Family Sciences, University of Texas at Austin, Austin, TX, United States of America; 3 Department of Psychology, University of California, Santa Cruz, CA, United States of America; 4 Department of Psychology, University College London, London, United Kingdom; South African Medical Research Council, SOUTH AFRICA

## Abstract

During the past 50 years, there have been marked improvement in the social and legal environment of sexual minorities in the United States. Minority stress theory predicts that health of sexual minorities is predicated on the social environment. As the social environment improves, exposure to stress would decline and health outcomes would improve. We assessed how stress, identity, connectedness with the LGBT community, and psychological distress and suicide behavior varied across three distinct cohorts of sexual minority people in the United States. Using a national probability sample recruited in 2016 and 2017, we assessed three a priori defined cohorts of sexual minorities we labeled the *pride* (born 1956–1963), *visibility* (born 1974–1981), and *equality* (born 1990–1997) cohorts. We found significant and impressive cohort differences in coming out milestones, with members of the younger cohort coming out much earlier than members of the two older cohorts. But we found no signs that the improved social environment attenuated their exposure to minority stressors—both distal stressors, such as violence and discrimination, and proximal stressors, such as internalized homophobia and expectations of rejection. Psychological distress and suicide behavior also were not improved, and indeed were worse for the younger than the older cohorts. These findings suggest that changes in the social environment had limited impact on stress processes and mental health for sexual minority people. They speak to the endurance of cultural ideologies such as homophobia and heterosexism and accompanying rejection of and violence toward sexual minorities.

## Introduction

For decades, researchers have demonstrated that sexual minority people experience disparities in multiple indicators of mental health and physical health when compared to nonsexual minority populations [1]. Minority stress theory has been a primary causal model explaining these health disparities. Minority stress builds on social and psychological theories about stigma and prejudice, social structures and health, and stress [2]. It describes how stressors that stem from prejudice and stigma, along with resilience resources (e.g., social support, community connectedness), affect the health of sexual and gender minority people [2–6].

Minority stress theory starts with the observation that stressors are not distributed randomly in society but are tied to social positions and social processes [7,8]. The basic premise of the theory is that all other things being equal (e.g., race and ethnicity, social class, gender), prejudice toward sexual minorities predisposes them to excess stress as compared with their heterosexual counterparts. In turn, this excess stress increases the risk of negative outcomes caused by stress and contributes to health disparities [2,9].

By its basic premise, minority stress is understood in social context: It is a social theory of stress and health. Therefore, we would expect that as social context shifts, so would experiences of minority stress and resultant health outcomes. Thus, if prejudice toward sexual minority people declined, minority stress and in turn, health disparities would decline. Indeed, during the past 50 years, there have been marked changes in the social environment of sexual minorities in the United States (and other societies). Younger cohorts of sexual minority people have been living in a world that in many ways was not imaginable to older cohorts of sexual minority people when they were young [10,11]. In this study, we tested the proposition that in the context of increasing societal acceptance and cultural inclusion of sexual minority people, younger cohorts of sexual minorities fare better than older cohorts in terms of exposure to stress and health.

### Shifting social environment for sexual minority people

One of the most consistently used measures related to attitudes toward sexual minorities is a question in the General Social Survey that has been asked since the 1970s. Data have shown that between 1973 and 2010, the proportion of Americans who said that homosexuality is “always wrong” declined from 70% to 43% [12]; by 2014, that proportion declined to 40% [13]. Attitudes toward same-sex marriage also show similar trends. In 1996, 27% of Americans said they thought “same-sex couples should be recognized by the law as valid, with the same rights as traditional marriages”. By 2015, the year the U.S. Supreme Court recognized same-sex marriage as constitutionally mandated, the proportion of Americans saying that marriage for same-sex couples should be valid had grown to 60% [14]. By 2018, public support for marriage for same-sex couples had grown to 67%, defying expectations that the court’s decision would lead to a backlash against same-sex marriages [14].

Other shifts in the social environment are even more pronounced. During the past 50 years, homosexuality was reconceptualized from a mental illness, as defined by the *Diagnostic and Statistical Manual of Mental Disorders* (DSM-I and II) in 1952 and 1968, to a normal variation of human sexual behavior, as it has been understood since the declassification of homosexuality as a mental disorder in 1973 [15–17].

In law, in 2003, the Supreme Court declared sodomy laws unconstitutional in *Lawrence v*. *Texas*, reversing a decision to affirm the constitutionality of sodomy laws just 17 years earlier (*Bowers v*. *Hardwick*, 1986). Today, more than 58% of the sexual minority population in the United States live in areas that are defined as fair or better because of the existence of state laws that protect sexual minority people, such as nondiscrimination statutes protecting sexual minority people from unfair treatment in employment and housing and the absence of laws that marginalize sexual minority people, such as laws that restrict child welfare or medical services to sexual minority people based on the provider’s religious objection [18].

Together, these changes have led to markedly different social environments for sexual minority people who came of age in the past 50 years. A person who came of age immediately after Stonewall—the 1969 New York City uprising that dramatically increased visibility of the modern gay rights movement [19]—grew up at a time when homosexuality was criminalized, considered a mental disorder, and rejected by most Americans. Sexual minority people who came of age in the 2000s can plan on getting married and having children. Younger cohorts of sexual minority people can imagine themselves as accepted by family and community. In high school, they may have had an opportunity to join with other sexual minority youth and allies in school activities (e.g., gender and sexuality alliances) [20]. With the availability of the internet, they can find resources online, no matter how far they are from a major urban center, where most LGBT resources concentrated in the past, if they were available at all. In national media (films, TV shows, newspaper and magazine articles), a sexual minority person who came of age after 2000 can see positive role models and a positive portrayal of life for sexual minorities.

Of course, this is an optimistic, perhaps idyllic, portrayal of the lives of young sexual minority people in the United States. In the context of rapid social change, the experiences of sexual minority people vary significantly based on their social or geopolitical setting. Although the Movement Advancement Project identified 58% of the sexual minority population as residing in a fair to positive policy environment, 42% of sexual minority people live in states defined as having a poor social environment by these measures, and 29 states still do not prohibit discrimination based on sexual orientation [21]. Recent studies showed that in the years before federal marriage equality (2015), state ballot initiatives to limit marriage were linked to the health and well-being of sexual minority adults [22–25] and homophobic bullying for school-aged students [26].

Regardless of the political climate, for many sexual minority youth, acceptance at home, in school, and in religious communities is still unattainable [27]. Additionally, studies have shown that even after equal marriage was passed, minority stressors still persist for same-sex couples, particularly in the familial domain [28–30].

### Has minority stress changed in the past few decades?

The life course of sexual minority people is characterized by some transitions that are distinct, such as awareness of sexual minority identity (e.g., lesbian, gay, bisexual, queer) and coming out, as well as normative life transitions, such as establishing healthy adult relationships, completing education, and beginning employment [31,32]. Because of the vast changes in the status of sexual minority people in our society during the past 50 years, each recent cohort of sexual minority people has faced a different environment in terms of legal status, community attitudes, health context, and parental and familial acceptance [33,34].

These contextual factors likely create divergent experiences for sexual minorities, but we know little about their impact on minority stress and health. We do not know how minority stress has been experienced by different cohorts of sexual minority people, but it is reasonable to expect that exposure to minority stress would have declined among younger sexual minority people [35,36]. Thus, we would expect that today, sexual minority people are less likely to be fired from a job because of their sexual orientation than they were in the 1970s, not only because it is illegal in many regions—in June 2020, the Supreme Court declared employment discrimination of sexual and gender minorities unconstitutional across the United States—but also because of greater social acceptance of sexual minority people. Similarly, sexual minority people in older cohorts often experienced rejection from family members, which has possibly declined for younger sexual minority people today.

By extension, internalizing, or proximal, minority stressors, such as internalized homophobia—a minority stressor that is a significant predictor of adverse health and well-being [37]—should be lower for younger sexual minority people if they experience more affirming and less rejecting attitudes then their older peers had experienced as youth. The same is true for expectations of rejection and discrimination and for concealing one’s sexual minority identity [2]. Despite being a stressor in its own right, concealing one’s sexual minority identity is often used as a protective mechanism, in an attempt to avoid rejection and violence. As the social environment improved, people might be less fearful of rejection and violence and therefore, have less of a need to conceal their sexual minority identity. Therefore, we asked: Have improved social attitudes and legal conditions led to significant differences in the experience of minority stress among members of cohorts of sexual minority people?

### Distinct cohorts of sexual minority people related to changes in the social environment

To address this question and test the impact of the changing social environment on the lives of sexual minority people, we conceptualized three distinct cohorts of sexual minority people that correspond to significant social changes in the U.S. regarding law, policy, and culture.

One way to think about the social environment of sexual minority cohorts is to consider the discourse about sexual minorities at the time the cohort members were young teens. What would have been the main themes people learned about had they, at that young age, connected with the sexual minority discourse? Using this heuristic, we defined the age of 10 years old—considered a significant age for sexual development [38]—plus or minus 3 years, as a significant developmental period with which to define our cohort. We then identified significant historical events that characterized the social environment of sexual minority people since 1969 (the full list is available on the study website at www.generations-study.com) and lined them up historically against the lifeline of people beginning at age 10.

We identify three events as anchors that characterized three periods in the lives of sexual minorities: the Stonewall uprising (1969), the formation of ACT UP (1987), and the Massachusetts Supreme Court ruling that it was unconstitutional to deny marriage to same sex couples (2003). Around these events, we defined three distinct cohorts. Table 1 provides a sample of historical events that characterize each cohort.

**Table 1:** Historical context for the definition of three cohorts of sexual minorities in the United States.

Pride Cohort Born 1956–1963		Visibility Cohort Born 1974–1981		Equality Cohort Born 1990–1997	
1967	First officially recognized gay and lesbian campus group is formed: Student Homophile League, Columbia University	1985	Names Project memorial quilt for AIDS victims launches	2002	Nevada voters approve constitutional amendment defining marriage between man and woman
1967	*The Advocate* begins publishing	1985	First school for openly gay and lesbian teens opens in NYC (Harvey Milk School)	2003	Massachusetts Supreme Court rules it unconstitutional to deny marriage to same sex couples
1967	First U.S. gay bookstore opens: Oscar Wilde Memorial Bookshop, Greenwich Village	1985	Artificial insemination becomes available to unmarried women (start of the lesbian “baby boom”)	2003	U.S. Supreme Court strikes down remaining sodomy laws (*Lawrence v*. *Texas*)
1968	APA moves homosexuality from “sociopathic” category to “sexual deviation”	1985	Rock Hudson comes out, admits he has AIDS	2003	Reuben Zellman becomes first openly transgender person accepted to Hebrew Union College–Jewish Institute of Religion
1969	Stonewall Inn Riots occur	1986	US Supreme Court rejects challenge to state sodomy laws, rules sodomy laws are constitutional	2004	Maine legalizes same-sex partnerships
1969	President Richard Nixon takes office	1987	ACT UP forms	2004	New Jersey legalizes same-sex partnerships
1969	Lesbian concerns are introduced into the National Organization for Women	1987	2nd National March on Washington occurs, Names Project quilt is shown	2004	President George W. Bush calls for constitutional amendment defining marriage between man and woman
1969	Evelyn Hooker (NIMH study) urges decriminalization of private sex acts between consenting adults	1988	National Coming Out Day launches	2004	Constitutional amendments defining marriage between man and woman pass in Missouri, Louisiana, Arkansas, Georgia, Kentucky, Mississippi, Montana, North Dakota, Ohio, Oklahoma, Oregon, and Utah
1970	First gay pride parade is held in New York	1988	City College of San Francisco approves creation of the first gay and lesbian studies department in the United States	2004	*The L Word* premieres on television
1970	Gay “zaps” begin, first against NYC Mayor John Lindsay	1990	First National Bisexual Conference is held in San Francisco	2005	Constitutional amendment defining marriage between man and woman passes in Texas and Kansas
1970	Unitarian Universalist Association becomes first US mainstream religious group to recognize LGB clergy and laity in its ranks and demands an end to antigay discrimination	1990	Federal Hate Crimes Statistics Act passes; first law extending federal recognition to gays and lesbians	2006	Kim Coco Iwamoto becomes first transgender official to win statewide office in Hawaii
1970	Vatican issues statement reiterating that homosexuality is a moral aberration	1990	US restriction against gay immigrants is lifted	2006	Constitutional amendment defining marriage between man and woman passes in Alabama, Colorado, Idaho, South Carolina, South Dakota, Tennessee, Virginia, and Wisconsin
1970	“Lavender Menace” protest occurs at a feminist conference, urging National Organization for Women to change its stance on lesbianism.	1990	BiNet USA and Queer Nation are founded as national LGBT activist organizations	2007	Candis Cayne plays Carmelita Rainer, a trans woman having an affair with New York attorney in *Dirty Sexy Money* (recurring trans character)
1971	University of Michigan establishes the first collegiate LGBT programs office, then known as the “Gay Advocate’s Office”	1990	Union for Reform Judaism announces national policy: “All Jews are religiously equal regardless of their sexual orientation”	2008	George Takei (*Star Trek*) marries Brad Altman
1972	First gay and lesbian delegates are sent to the Democratic Convention	1991	First Black Lesbian and Gay Pride celebration is held in Washington, DC	2008	Oregon legalizes same-sex partnerships
1972	East Lansing, MI becomes first city to ban antigay bias in hiring	1991	Amnesty International decides to work on behalf of those imprisoned for consensual same-sex acts	2008	Connecticut legalizes same-sex marriage
1972	Washington Supreme Court rules that teachers can be fired for being homosexual (review is denied by U.S. Supreme Court in 1977)	1991	Red ribbon is first used as a symbol of the campaign against HIV/AIDS	2008	California Proposition 8 passes (voters approve constitutional amendment defining marriage between man and woman)
1973	APA removes homosexuality from list of mental illnesses	1991	Three same-sex couples file suit seeking right to marry in Hawaii	2008	Constitutional amendment defining marriage between man and woman passes in Arizona and Florida
1974	Lesbian Herstory Archives open to public	1991	First lesbian kiss on television occurs (*L*.*A*. *Law*)	2008	Ellen DeGeneres marries Portia de Rossi

We named each cohort to capture that cohort’s essential characteristics. We described the first, older cohort (of people born 1956–1963), as the *pride* cohort. These individuals came of age at a time when homosexuality was considered a mental disorder and sodomy was illegal in many states. But sexual minority people in this era began early efforts to cultivate pride in their communities. It was an era when sexual minority (a “gay” identity) was born as a modern civil rights movement, with slogans such as “gay is good” and the first gay pride march [39].

We named the second cohort the *visibility* cohort (born 1974–1981). The main discourse when they were experiencing puberty was around the AIDS epidemic that particularly affected men who have sex with men but had a profound impact on the entire LGBT community [40]. Increasing visibility of sexual minorities portrayed them both as good, as caretakers of their own and activists, but also as bad—as victims of a disease they deserved. When gay and bisexual men of this cohort became sexually active in the 1990s and 2000s, HIV/AIDS had already become an epidemic. This cohort of men benefited from better HIV prevention education and improved AIDS antiretroviral therapies. These therapies changed HIV from the death sentence it had been just a cohort before to a dreaded, but manageable, disease [40]. One of the hallmarks of this period was a significant strengthening of LGBT institutions including political activist organizations, community health centers, and other community development [41]. Among many milestones, this period saw the establishment of an LGBT high school in New York City, the National March on Washington, and the first gay and lesbian studies department established in City College of San Francisco.

We named the youngest cohort the *equality* cohort (born 1990–1997). The discourse during the period of their teenage years had shifted to a discourse about the equality of sexual minorities and demands (and some successes) regarding their cultural inclusion. The discourse around equality was most resonant (and successful) around marriage equality. Members of this cohort witnessed reversal of the federal “Don’t Ask, Don’t Tell” policy that prohibited sexual minority military personnel to be open about their sexual identities and the invalidation of significant parts of the Defense of Marriage Act by the Supreme Court. Also, significantly, public attitudes in the United States changed to reflect more positive views of homosexuality [42,43]. Thus, sexual minority people in the equality cohort grew up in a less prejudicial society as compared with the older cohorts.

Using a probability sample of sexual minorities in the United States, we asked: How do these three cohorts differ in levels of minority stress experiences, their association with LGBT community, and mental health outcomes like distress and suicidality? Answering these questions is significant to understanding the causes of health disparities, developing public health and policy interventions, and improving clinical interventions with sexual minority people across the life course.

## Materials and methods

### Sample

Data were collected as part of the Generations study, a 5-year study designed to examine health and well-being across three cohorts of sexual minority people. The Generations study used a two-phase recruitment procedure. In the first phase, utilizing a question asked of all Gallup respondents in the Daily Tracking Survey (see Measures), all LGBT individuals were identified. In the second phase, respondents who identified as LGBT were assessed for eligibility for participation in the Generations study, and those eligible were invited to participate in the survey [44].

The first phase was conducted by Gallup using a national probability sample of adults aged 18 or older. Gallup used a dual-frame sampling procedure, which included random-digit dialing to reach both landline and cellphone users. Gallup stratified the dialing list to ensure that the unweighted samples were proportionate by U.S. census region and time zone. Gallup weighted the data daily to compensate for disproportionalities in nonresponse and selection probabilities.

The second phase consisted of a self-administered survey. Respondents were eligible if they identified as cisgender or a gender nonbinary sexual minority; belonged to the three cohorts under investigation (aged 18–25, 34–41, or 52–59); were Black, Latino, or White; completed sixth grade at least; and spoke English well enough to conduct the phone interview in English. (Respondents who identified as transgender, regardless of their sexual orientation, were screened for participation in a related *TransPop* study).

Eligibility was limited to these age groups because the scientific focus concerned differences among age cohorts related to the social environment, as previously described. Eligibility was limited to the three larger racial and ethnic groups in the United States, as well as bi- or multiracial groups that included one of these, because estimates of recruitment showed that we would not be able to get a sufficient number of respondents who belonged to smaller racial and ethnic groups (i.e., Asian and Native American or Alaska Native). Eligibility was restricted to sixth grade or higher education to ensure reading comprehension for self-administration of the survey.

Eligible respondents who agreed to receive the Generations survey received a survey questionnaire by email or mail to complete by self-administration (via a web link or printed questionnaire, respectively). Respondents received a $25 gift certificate together with their survey materials. Prior to completing the survey, respondents reviewed the consent information. Respondents who received the survey electronically via email submitted the web survey online; respondents with mailed surveys returned the questionnaires using a provided preaddressed, prestamped envelope.

Recruitment lasted a year, between March 28, 2016, and March 30, 2017, with recruitment of 366,644 participants screened by Gallup. Of them, 3.5% identified as LGBT, and 3,525 met eligibility criteria. Of those eligible, 81% agreed to participate in the survey and of those, 48% completed the survey, for an overall cooperation rate of 39%. To increase the number of racial and ethnic minority respondents, we oversampled Black and Latino respondents using the same procedures by extending the recruitment period (April 1, 2017, to March 30, 2018). The final Generations baseline sample size was 1,518, including 1,331 from the original sample and 187 from the enhancement sample.

The study was approved by the IRBs of UCLA and Gallup. The UCLA IRB served as the lead IRB for collaborating universities. Respondents were anonymous to the investigators, information about respondents’ identity was held by Gallup, which administered the survey. In lieu of signed consent, respondents received an information sheet that they had to accept prior to participating in the survey.

### Measures

#### Sexual identity

In Phase 1, Gallup asked all respondents: “Do you, personally, identify as lesbian, gay, bisexual, or transgender?” with response options “yes, do” or “no, do not”. Because this question includes both LGB and transgender people, we followed up with people who said “yes”. To assess sexual minority status, respondents were asked, “Which of the following best describes your current sexual orientation?” with the response options, “straight/heterosexual”, “lesbian”, “gay”, “bisexual”, “queer”, “same-gender loving”, or “other”; if they answered that they were not heterosexual, they were considered a sexual minority person.

#### Gender identity

In Phase 2, respondents were also asked two questions to determine their gender identity: First, “On your original birth certificate, was your sex assigned as female or male?” with the response options of “female” or “male”, and then, “Do you currently describe yourself as a man, woman, or transgender?” with the response options of “man”, “woman”, and “transgender”. Respondents who said they were transgender were then asked, “Are you trans woman (male-to-female), trans man (female-to-male), or nonbinary or genderqueer”. Respondents were classified as transgender if they said they were transgender in the second question or if their current gender identity (second question) was different than their sex assigned at birth (first question).

#### Coming out milestones

Respondents reported the age when they were first sexually attracted to a same-sex person, age of first sexual relationship, age of first intimate same-sex relationship, age when they first realized they were LGB, and age when they first told a family member that they were LGB.

#### Minority stressors

*Sexual orientation change effort*. Also referred to as conversion therapy, this measure was developed by the Generations investigators. Respondents were asked, “Did you ever receive treatment from someone who tried to change your sexual orientation (such as try to make you straight/heterosexual)”. Responses coded as having ever received such treatment or not.

*Victimization*. These experiences were assessed using six items that asked whether the respondents experienced during their lifetime being physical and sexual assaulted, threatened with assault, and verbally abused [45]. Responses were dichotomized into “once or more” versus “never”.

*Everyday discrimination*. This 9-item scale assesses common experiences of microaggression, including being disrespected, treated unfairly, and harassed over a year prior to the interview [46]. Responses were recorded on a 4-point Likert scale ranging from “often” to “never”. The scale score was created as a mean score of each item within the scale. The resulting variable was reverse-coded so that lower values represented less everyday discrimination and higher values represented more everyday discrimination. Scale values ranged from 1 to 4 (Cronbach’s alpha = .91).

*Felt stigma*. This 3-item scale developed by Herek [45] asks about respondents’ expectations that they will be stigmatized by others in their community, asking whether “most people” where the respondent lives would think less of or be less likely to hire someone who is openly LGB. Responses were recorded on a 5-point Likert scale ranging from “strongly disagree” to “strongly agree”, with a middle category of “neither agree nor disagree”. Lower values represented less felt stigma and higher values represented greater felt stigma. Scale values ranged from 1 to 5 (Cronbach’s alpha = .70).

*Internalized homophobia (revised)*. This 5-item scale assesses the respondent’s feelings about being a sexual minority, including whether the respondent tried to stop being attracted to people of the same sex, felt that being sexual minority is a shortcoming, or sought professional help to change their sexual orientation to become straight [47]. Responses were recorded on a 5-point Likert scale ranging from “strongly disagree” to “strongly agree”. The scale score was created as a mean score of each items in the scale. Lower values represented less internalized homophobia and higher values represented greater internalized homophobia. Scale values ranged from 1 to 5 (Cronbach’s alpha = .75).

#### Mental health indicators

*Psychological distress*. This 6-item scale asks how often, in the past 30 days, respondents felt “nervous”, “hopeless”, “restless or fidgety”, “so depressed that nothing could cheer you up”, “that everything was an effort”, and “worthless” [48]. Responses were recorded on a 5-point scale ranging from “all of the time” to “none of the time” (Cronbach’s alpha = .89).

*Suicide attempt*. This was assessed with the Army Study to Assess Risk and Resilience in Service Members instrument [49]. Respondents answered “yes” or “no” to a series of questions including, “Did you ever make a suicide attempt (i.e., purposefully hurt yourself with at least some intention to die)?”

#### Identity and LGBT community affiliation

*Connection with the LGBT community*. This 7-item scale, adapted from the 8-item scale described by Frost and Meyer [50], assesses the desire for and strength of LGBT community affiliation among respondents. Scale items include “You feel you’re a part of the LGBT community” and “You are proud of the LGBT community”. Responses were recorded on a 4-point Likert scale ranging from “agree strongly” to “disagree strongly”. The scale variable was created as a mean score of each item in the scale. The final scale was reverse-coded so that lower scores represented lower community connectedness, whereas higher scores represented greater community connectedness. Scale values ranged from 1 to 4 (Cronbach’s alpha = .86).

*Sexual identity centrality*. This 5-item subscale from Mohr and Kendra’s [51] 27-item Lesbian, Gay, and Bisexual Identity Scale assesses the degree to which respondents’ sexual identities were central to their overall identities. Scale items include “My sexual orientation is an insignificant part of who I am” and “Being an LGB person is a very important aspect of my life”. Responses were recorded on a 6-point Likert scale ranging from “disagree strongly” to “agree strongly”. The scale score was created as a mean score of each item in the scale. Lower values represented lower centrality and higher values represented greater centrality. Scale values range from 1 to 6 (Cronbach’s alpha = .81).

### Statistical analyses

We used descriptive statistics to describe demographic characteristics of the sample (Table 2). We calculated point estimates and 95% confidence intervals to assess differences among the cohorts in lieu of significance testing because we did not pose specific hypotheses regarding the demographics of the cohorts. In Table 3, we reported differences among the cohorts in eight categorical and six continuous outcome measures. For the eight categorical variables, we present point prevalence estimates and 95% confidence intervals weighted for the sampled population. We assessed differences among the cohorts using odds ratios (OR) and 95% confidence intervals. For the six continuous variables, we present means and standard deviations and tested differences among the cohorts using the *F*-test and indicate *p*-values less than .05.

**Table 2:** Select demographic characteristics in generations study by cohort, estimated population percentage and 95% confidence interval (*N* = 1,518).

	Total Sample	Equality Cohort (ages 18–25)	Visibility Cohort (ages 34–41)	Pride Cohort (ages 52–59)
	(*N* = 1,518)	(*n* = 670)	(*n* = 372)	(*n* = 476)
	% (95% CI)	% (95% CI)	% (95% CI)	% (95% CI)
Gender				
Woman	55.01 (51.90, 58.07)	59.02 (54.69, 63.22)	55.95 (49.87, 61.86)	39.61 (34.70, 44.73)
Man	37.58 (34.70, 40.56)	31.15 (27.38, 35.20)	40.53 (34.74, 46.59)	56.88 (51.71, 61.89)
Gender nonbinary	7.41 (5.85, 9.35)	9.82 (7.45, 12.84)	3.52 (2.15, 5.72)	3.52 (2.01, 6.09)
Sexual identity				
Straight	1.32 (0.67, 2.60)	1.22 (0.48, 3.09)	2.04 (0.61, 6.60)	0.82 (0.18, 3.677)
Lesbian	17.26 (15.05, 19.71)	14.54 (11.66, 17.98)	17.46 (13.17, 22.79)	26.65 (22.39, 31.39)
Gay	29.03 (26.42, 31.79)	21.47 (18.16, 25.19)	31.76 (26.60, 37.41)	52.60 (47.43, 57.71)
Bisexual	40.02 (36.91, 43.22)	47.4 (43.01, 51.83)	39.29 (33.36, 45.55)	14.70 (11.37, 18.81)
Queer	5.72 (4.51, 7.23)	7.05 (5.30, 9.33)	5.92 (3.84, 9.02)	0.77 (0.30, 1.98)
Same-gender loving	1.17 (0.74, 1.83)	0.49 (0.16, 1.45)	1.41 (0.63, 3.10)	3.29 (1.82, 5.86)
Asexual	1.66 (0.95, 2.87)	2.44 (1.35, 4.40)	0.48 (0.11, 2.10)	0.23 (0.04, 1.85)
Pansexual	3.32 (2.35, 4.67)	4.819 (3.32, 6.95)	1.65 (0.75, 3.60)	0
Anti-label	0.23 (0.06, 0.83)	0.23 (0.03, 1.60)	0	0.50 (0.14, 1.72)
Something else	0.28 (0.08, 0.94)	0.34 (0.07, 1.56)	0	0.42 (0.09, 1.93)
Education				
High school or less	42.51 (39.23, 45.85)	54.59 (50.32, 58.80)	23.51 (17.54, 30.77)	22.38 (17.57, 28.07)
Race and ethnicity				
White	62.22 (59.15, 65.20)	56.59 (52.22, 60.85)	65.15 (59.24, 70.63)	78.69 (73.92, 82.79)
Black	16.52 (14.37, 18.93)	17.75 (14.76, 21.2)	18.02 (13.89, 23.05)	10.36 (7.44, 14.25)
Latino	21.26 (18.81, 23.94)	25.66 (22.10, 29.58)	16.83 (12.89, 21.66)	10.95 (8.01, 14.81)
Urbanicity				
Urban	87.28 (84.95, 89.29)	86.74 (83.36, 89.52)	88.46 (83.61, 92.01)	87.77 (84.04, 90.72)
Region				
Northeast	18.97 (16.68, 21.50)	16.95 (13.95, 20.44)	22.63 (17.89, 28.19)	21.76 (17.64, 26.53)
Midwest	20.09 (17.60, 22.82)	20.57 (17.18, 24.42)	21.88 (16.78, 28.00)	16.23 (12.85, 20.30)
South	34.64 (31.69, 37.71)	37.08 (32.90, 41.45)	29.11 (23.98, 34.84)	32.62 (28.03, 37.58)
West	26.30 (23.69, 29.10)	25.40 (21.79, 29.40)	26.38 (21.63, 31.76)	29.39 (24.92, 34.29)
Unemployed	8.30 (6.46, 10.60)	10.67 (8.00, 14.08)	6.15 (3.27, 11.28)	2.67 (1.32, 5.33)
Poverty (200% poverty level or worse)	41.64 (38.48, 44.88)	48.05 (43.61, 52.52)	36.17 (30.10, 42.72)	25.41 (20.86, 30.57)
Own home	25.68 (23.26, 28.25)	11.91 (9.26, 15.20)	34.08 (28.88, 39.70)	64.24 (58.96, 69.20)

**Table 3:** Exposure to minority stressors, sexual orientation affiliation, and mental health outcomes by cohort (N = 1,518).

	Cohort 1 (Equality)	Cohort 2 (Visibility)	Cohort 3 (Pride)	Cohorts 1 vs. 2	Cohorts 1 vs. 3	Cohorts 2 vs. 3
	% (95% CI)	% (95% CI)	% (95% CI)	*OR* (95% CI)	*OR* (95% CI)	*OR* (95% CI)
*Categorical variables*						
Sexual orientation change effort (lifetime)	6.15 (4.32, 8.67)	8.30 (5.37, 12.62)	7.79 (5.33, 11.25)	0.98 (0.94, 1.02)	0.98 (0.95, 1.02)	1.01 (0.96, 1.05)
Hit, beaten, physically attacked, sexually assaulted since age 18 (once or more)	37.33 (33.13, 41.74)	51.51 (45.36, 57.61)	42.85 (37.77, 48.09)	0.87 (0.81, 0.94)	0.95 (0.89, 1.01)	1.09 (1.01, 1.18)
Robbed or property was stolen, vandalized, or purposely damaged since age 18 (once or more)	28.90 (25.07, 33.05)	55.20 (48.99, 61.26)	64.90 (59.77, 69.70)	0.77 (0.71, 0.83)	0.70 (0.66, 0.74)	0.91 (0.84, 0.98)
Someone tried to attack you, rob you, or damage your property, both didn’t succeed since age 18 (once or more)	17.56 (14.37, 21.28)	29.00 (23.69, 34.97)	30.67 (25.94, 35.85)	0.89 (0.84, 0.95)	0.88 (0.83, 0.93)	0.98 (0.91, 1.06)
Someone threatened you with violence since age 18 (once or more)	46.08 (41.69, 50.53)	61.38 (55.37, 67.06)	55.59 (50.39, 60.68)	0.86 (0.80, 0.92)	0.91 (0.85, 0.97)	1.06 (0.98, 1.15)
Someone verbally insulted or abused you since age 18 (once or more)	72.14 (68.02, 75.92)	80.74 (75.62, 84.99)	77.78 (73.26, 81.73)	0.92 (0.86, 0.98)	0.95 (0.89, 1.00)	1.03 (0.97, 1.10)
Someone threw an object at you since age 18 (once or more)	34.55 (30.44, 38.91)	51.79 (45.67, 57.86)	41.88 (36.79, 47.14)	0.84 (0.78, 0.91)	0.93 (0.87, 0.99)	1.10 (1.02, 1.20)
Suicide attempt (lifetime)	30.45 (26.49, 34.72)	24.29 (19.48, 29.84)	21.01 (16.90, 25.81)	1.06 (0.99, 1.14)	1.10 (1.03, 1.17)	1.03 (0.97, 1.11)
*Continuous variables*	*M* (*SD*)	*M* (*SD*)	*M* (*SD*)	*F*	*F*	*F*
Everyday discrimination	2.152 (0.033)	2.001 (0.048)	1.638 (0.032)	6.86**	128.32***	40.23***
Felt stigma	2.718 (0.042)	2.693 (0.054)	2.699 (0.051)	0.14	0.08	0.01
Internalized homophobia	1.707 (0.032)	1.649 (0.050)	1.514 (0.034)	0.96	17.22***	4.99*
Connection with the LGBT community	3.034 (0.026)	2.860 (0.044)	2.916 (0.029)	11.67**	9.32**	1.14
Sexual identity centrality	3.932 (0.047)	3.808 (0.076)	4.019 (0.060)	1.93	1.29	4.75*
Psychological distress	10.176 (0.241)	7.669 (0.372)	5.364 (0.257)	32.03***	186.45***	25.96***

*Note*. Results presented used analysis for weighted survey research; CI = confidence interval, OR = odds ratio, SD = standard deviation.

*p < .05.

**p < .01.

***p < .001.

## Results

In Table 2, we show demographic characteristics of the total sample and the three cohorts. Results show that more people in the younger equality cohort than the two older cohorts were gender nonbinary and more people in the older pride cohort were men. Younger cohort people were also more likely to use terms other than lesbian, gay, and bisexual to identify themselves (including queer and pansexual). As expected, younger people had lower education levels, were more likely to be unemployed and poor, and less likely to own a home than older people. Older people were also more likely than members of the two younger cohorts to be White.

### Coming out milestones

**Fig 1:**
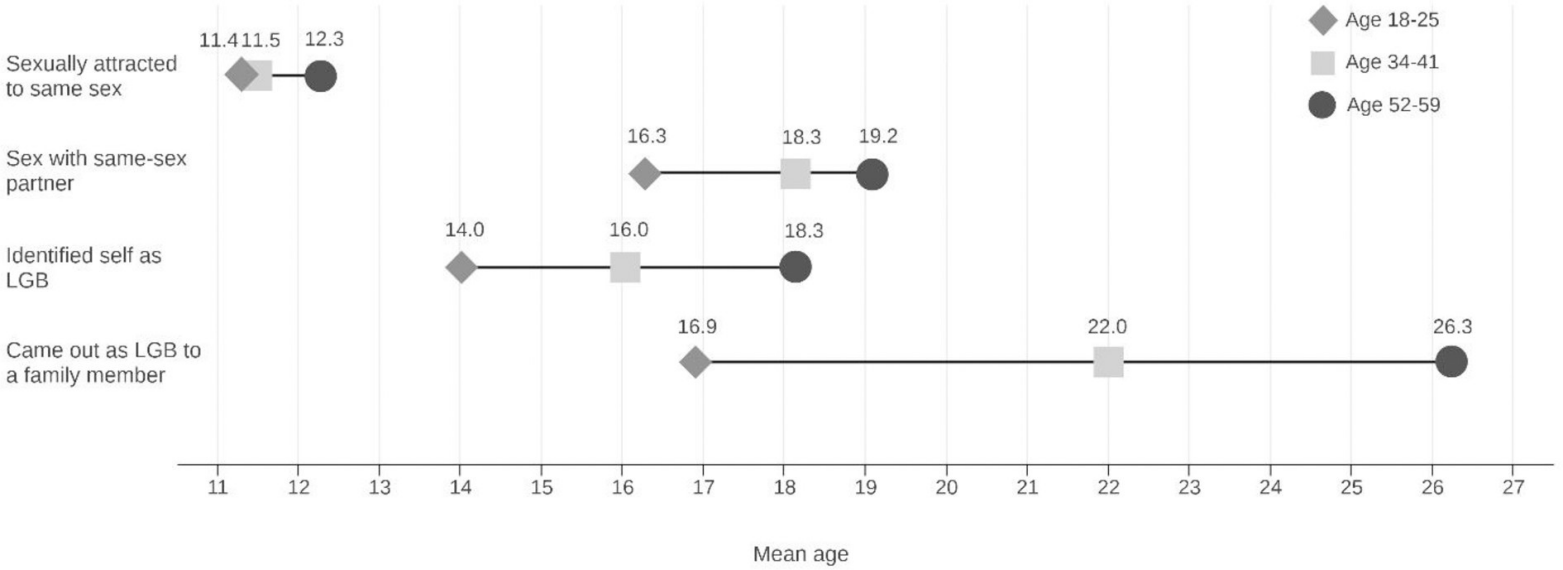
Ages at milestones related to LGB attraction, behavior, identity, and disclosure by age cohort, (N = 1,518).

Fig 1 shows ages at milestones related to same-gender attraction, behavior, identity, and disclosure by cohort. The figure shows that age when first sexually attracted to a person of the same gender was about the same across the cohorts (*M* = 11.6 years, *SE* = 0.15, Wald test *F* = 2.11, *p* = .12). But there were significant age differences for every other milestone, with the younger cohort of LGB people having earlier onset of a sexual relationship, realizing they were LGB, and telling a family member they were LGB (*F* = 21.77, *p* < .001; *F* = 45.83, *p* < .001; *F* = 195.35, *p* < .001, respectively).

### Minority stressors

In Table 3, we show differences and similarities among cohorts in exposure to minority stressors, community affiliation, and mental health indicators. There were small, nonsignificant differences in the proportions of people who had been subjected to sexual orientation change effort in their lifetime across cohorts, with about 6% to 8% of all sexual minority people experiencing sexual orientation change effort and ORs indicating statistical nonsignificance, hovering around 1.

In terms of lifetime experiences of victimization (since age 18), we found that more sexual minorities in the older or middle cohorts than in the younger cohort reported having been hit, beaten, physically attacked, or sexually assaulted (*F* = 8.27, *p* < .001), with statistically significant differences between the younger and middle cohorts but not the younger and older cohorts or the middle and older cohorts. Results were significant for having been robbed or having had property stolen, vandalized, or purposely damaged, with all cohorts significantly different from one another, showing that the younger cohort had less exposure than the middle and older cohorts and the middle cohort had less exposure than the older cohort. The rest of the victimization items in Table 3—attempted robbery, having been threatened with violence, having been verbally insulted or abused, and having had an object thrown at—all had similar patterns, with statistically significant differences between the younger and middle and younger and older cohorts, showing that the younger cohort had less exposure than the middle and older cohorts; however, the differences between the middle and older cohorts were not statistically significant. (The difference between the younger and older cohorts for having been verbally insulted or abused was in the same direction but marginal in significance).

Sexual minority people in the younger cohort had experienced more everyday discrimination than sexual minority people in the middle and older cohorts, with the middle cohort having intermediate levels of everyday discrimination. In terms of internalized homophobia, the younger cohort did not differ from the middle cohort, but both the younger and middle cohorts had higher levels of internalized homophobia than the older cohort. The three cohorts did not differ in level of felt stigma (i.e., expectations of rejection and discrimination).

### Identity centrality and affiliation with the LGBT community

We assessed similarities and differences among the three cohorts in centrality of minority sexual identity and connection with LGBT community. On both measures, the mean scores were similar across the cohorts, but young cohort members reported the highest levels on both scales. The difference was significant for connection with the LGBT community between the younger and middle cohorts and the younger and older cohorts, but not between the middle and older cohorts. The difference in identity centrality of sexual orientation identity was significant only between the middle and older cohorts, with the older cohort having a slightly higher mean score on the scale (Table 2).

### Psychological distress and suicide

When it comes to psychological distress, members of the younger cohort reported higher levels of distress than both the middle and older cohorts, and the middle cohort reported a higher level of distress than the older cohort.

Regarding lifetime suicide attempts, 30% of the younger cohort, 24% of the middle cohort, and 21% of the older cohort reported at least one suicide attempt (only the difference between the younger and older cohorts was statistically significant).

## Discussion

We started this project with the hypothesis that younger cohorts of sexual minority people would fare better than their older peers, who grew up in a more hostile social and legal environment than that of the younger cohorts. We found a strong cohort impact on the age of same-sex attraction milestones: Each successive cohort had earlier sexual identity milestone experiences of identifying as a sexual minority person, first sexual experience, and coming out. This likely indicates both greater comfort in coming out and shifting social norms around sexuality and youth. On one hand, these trends suggest that the younger cohorts reached developmental milestones related to their sexuality earlier than older cohorts, which is generally understood to be positive for adjustment. On the other hand, identifying and coming out as a sexual minority can confer risk, including greater exposure to minority stressors and victimization [52].

Indeed, contrary to our hypothesis, we found little evidence that social and legal improvements during the past 50 years in the status of sexual minority people have altered the experiences of sexual minority people in terms of exposure to minority stressors and resultant adverse mental health outcomes. Most tellingly, younger sexual minority people did not have less psychological distress or fewer suicide attempts than older sexual minority people.

Regarding minority stress, we found that members of the younger cohort did not experience less minority stress than members of older cohorts. This was consistent across both distal minority stressors, which measure direct exposure to external conditions, such as antigay violence, and proximal stressors, which measure how homophobia is internalized and learned. Members of the younger cohort did experience fewer of the victimization experiences we studied. But the measure of lifetime exposure to victimization presents a challenge. By their nature, lifetime measures would show higher prevalence among older people simply because they have more years in their lifetime and therefore, more opportunities for experiencing victimization. It this context, it is notable that the younger sexual minority people experienced more extreme victimization in their shorter lifespan. More than 1 in 3 (37%) experienced being hit, beaten, physically attacked, or sexually assaulted; almost half (46%) had someone threaten them with violence; and almost 3 in 4 (72%) were verbally insulted or abused. In terms of proximal minority stressors—internalized homophobia and felt stigma—we found members of the younger cohort recorded as high or higher levels of stress relative to their older counterparts.

Consistent with findings on the experience of minority stressors, we found high scores of psychological distress in the younger cohort. Although some research has suggested that this may be a general trend for younger adults to have higher levels of depressive symptoms, there appears to be a U-shaped relationship in the general population, with younger and older people exhibiting high levels of depressive symptoms measured by the same scale we used [53]. We found a clear disadvantage to the younger cohort that seems unique to sexual minority people. Research has also shown that no significant bias in reporting patterns to this scale could explain the pattern of our results [54]. We also found that 30% of members of the younger cohort had attempted suicide. This is an alarming figure that was even higher than the high proportions of lifetime suicide attempts reported by the middle and older cohorts. By comparison, the proportion of young people aged 18–24 in the general population who have attempted suicide has been less than 4% [55].

Our findings are clearly inconsistent with the hypothesis. We started our hypothesis from a theoretical perspective that suggests that as social conditions improve, exposure to minority stressors and mental health problems would decrease. Our hypothesis was optimistic, but we were not blind to evidence to the contrary. As Russell and Fish [56] have shown, disparities by sexual identity have not been declining, but instead increasing. Most foretelling has been findings by the Centers for Disease Control and Prevention about exposure to stress among youth in high schools. Reports have consistently indicated that sexual minority youth experience significantly more stressful experiences than heterosexual youth and suffer significantly greater adverse health outcomes, including suicide ideation and attempts [57–60]. Our findings, thus, are consistent with studies that showed that minority stress and health disparities based on sexual orientation have not dissipated [56,61–64], despite the significant social and legal gains of the last decades.

Finally, contradicting writings about the declining significance of the LGBT community and sexual minority identity for the young cohort of sexual minority people, we found as high a sense of centrality of sexual minority identity and sense of connection with the LGBT community [35,36]. This is an important finding because it suggests that the LGBT community is still an important locale for connecting with LGBT identities, values that denounce homophobia, and role models for healthy sexual minority lives. As has been shown with older cohorts of sexual minorities, these are important resilience factors that allow sexual minority people to grow and overcome homophobia [2,65–69]. Connection with the LGBT community is also important for health information and the public health of LGBT communities, because resources serving sexual minorities have been organized under the LGBT banner for decades [70]. Studies have shown, for example, that gay and bisexual men who were connected to LGBT health resources were more likely than those who were not to use preexposure prophylaxis as HIV prevention [40]. However, this should not obscure the many challenges facing LGBT community organizers to overcome intracommunity rejection across race, social class, and other attributes [71].

There are many reasons why our hypothesis was not supported, and it is beyond our scope to explore these. Our approach was to examine cohort-wide patterns of change. In that, we may have missed the impact on specific segments of the populations. For example, we do not know whether White sexual minority people fared differently than ethnic minorities or how gender impacted the patterns we studied. This was, of course, purposeful because our theory was that the entire cohort would be affected by historical changes (even if not in equal ways). Also, it is plausible that social conditions, looked at as broadly as we did, do not reveal many other influences on stress exposure and mental health outcomes. For example, even if the social environment improved overall, it may have not improved in all microenvironments. Furthermore, it is possible that even as the social environment improves, the lived experience of sexual minority people continues to be challenging [72]. For example, a gay or lesbian teenager may be more accepted now than their older cohort peers had been when they were teenagers, but they were still a minority in their high school, deprived of opportunities for developing intimate relations. Also, a “developmental collision” may occur as sexual minority identity disclosure at younger ages coincides with normative developmental processes associated with adolescence [56]. Although the larger social context may have improved in such a way that emboldens younger generations to be out, the normative developmental context of adolescence remains one in which conformity is prized. Compulsions to conform to gender and sexual norms that privilege heterosexuality may continue to characterize adolescence in the United States [73]. Future analysis could determine whether some segments of the population benefited more than others from the improved social conditions and how improved social conditions impact the lived experience of sexual minority people.

### Study limitations

Our study was limited in several important ways that are relevant to drawing conclusions about cohort differences. First, our purpose was to provide an overview of the status of stress and health in three cohorts of sexual minority people at one point using cross-sectional data. Obviously, this one-time picture limits our ability to discuss historical differences and trajectories, but we interpret the results to suggest that they reflect the impact of historical changes in the status of sexual minority people in society. Our interpretation is based on theory and our a priori categorization of the three cohorts. Because we aimed to capture the impact of historical context, we erred by ignoring potential differences among members of any age cohort that could have affected variability in cohorts. We assessed differences among three cohorts of sexual minority people but not differences by gender, race and ethnicity, socioeconomic status, neighborhood context, etc. This is consistent with our hypothesis about cohort differences. Regardless of variability in each cohort, we tested the hypothesis that the younger cohort, as a whole, fared better than older cohorts because members of the young cohort, across all strata, enjoyed better social conditions than members of older cohorts.

Second, like all measures, our measures of stress, coping, and health were limited in that each measure has its limitations and represents only a portion of complex constructs. For example, we assessed depressive symptoms and suicide attempts as proxies for the construct of mental health. Nonetheless, we present a variety of stress measures that include victimization and everyday discrimination, internalized minority stressors (felt stigma and internalized homophobia), and generalized distress, which is associated with mental health and suicide attempts—a clear and serious outcome and significant gauge of sexual minority health. The two measures that represent resilience assessed connection with the community and centrality of identity—two important elements of coping with minority stress.

Third, cohort (and the historical periods of interest) and age were confounded. That is, there was no way to avoid the fact that respondents who came of age in more distant historical periods are also older than respondents who grew up in the context of recent and improved social conditions. Therefore, it is plausible that some differences that we observed resulted from developmental or age-related changes rather than the impact of the different historical social environments. For example, internalized homophobia typically is expected to decline with age, as a person comes to terms with their same-sex attraction and comes out [32]. Our finding that internalized homophobia was higher in the younger than older cohort is consistent with that theory and could reflect the younger developmental stage of the younger cohort members. On the other hand, if social conditions have improved so greatly, we could have expected that internalized homophobia—which denotes rejection of oneself because of one’s same-sex attraction and identity—would cease to be an issue for younger people altogether. That is definitely not the case. Our findings show that some younger people still struggle with self-acceptance. So, although we cannot say with certainty that there is no age effect, we certainly can say that internalized homophobia has not ended in young sexual minority people.

## Conclusion

Our study has many strengths. It is the first probability sample to provide nationally representative statistics on the specific experiences of sexual minority people using measures that were tailored to this population (as compared with general population surveys that did not include sexual minority-specific measures). It is the also the first large-scale study with a design that allows for inferences about the relationships between key health outcomes and social context among sexual minority populations.

Few social issues have shifted as dramatically during a half-century as cultural attitudes and social policies affecting sexual minorities. In the span of 50 years since the Stonewall uprising, homosexuality was declassified as a mental illness and eventually decriminalized. The community endured a large-scale public health epidemic and successfully advocated for rights and recognition, including the right to marry and most recently, protection against employment discrimination. Given the marked changes in the social and political contexts in the United States and elsewhere, it is difficult to imagine a uniform experience of development for sexual minorities. Rather, we would expect to find variability across cohorts in critical aspects of development. The evidence of changes in identity development processes, including opportunities for self-labeling and the timing of milestones, is clear [73–77]. But analysis of stress exposure and mental health suggests little distinction in the experience of minority stress across cohorts, indicating no discernable improvement in minority stress and health of sexual minorities.

These findings indicate the extent to which changes in the social environment have been limited in their impact on stress processes and mental health for sexual minority people. They speak to the endurance of cultural ideologies such as homophobia and heterosexism accompanying rejection of and violence toward sexual minorities. They call our attention to the continued need to recognize threats to the health and well-being of sexual minority people across all ages and remind us that LGBT equality remains elusive [78].

## Supporting information

S1 File (PDF)
