# Supplementary material for: Minority stress, distress, and suicide attempts in three cohorts of sexual minority adults: A U.S. probability sample
Source: PLoS One. 2021 Mar 3;16(3):e0246827. doi: 10.1371/journal.pone.0246827 (PMC7928455; doi:10.1371/journal.pone.0246827)
Supplement: S1 File — (PDF) [file pone.0246827.s001.pdf]

Generations Study Baseline Measures
Source document

Construct	Question # in Baseline	Source
**Positive Health**		
Cantril Scale	Q1-Q2	Hadley Cantril, 1965/ Gallup Poll. Retrieved from: http://www.gallup.com/poll/122453/Understanding-Gallup-Uses-Cantril-Scale.aspx
Happiness	Q3	PEW Research Center (2013)-A Survey of LGBT Americans. Retrieved from: http://www.pewsocialtrends.org/files/2013/06/SDT_LGBT-Americans_06-2013.pdf
Social Wellbeing	Q4-Q18	Keyes, Corey Lee M. (1998). Social Well-Being. *Social Psychology Quarterly, 61* (2) 121-140.
Satisfaction with life	Q186-Q190	Diener, E., Emmons, R. A., Larsen, R. J., & Griffin, S. (1985). The Satisfaction with Life Scale. *Journal of Personality Assessment, 49*, 71-75.
**Identity**		
Sex assigned at birth	Q27	Part one of a two-step approach on gender identity. The GenIUSS Group (2014). *Best practices for asking questions to identity transgender and other gender minority respondents on population-based surveys.* J.L. Herman (Ed.). Los Angeles, CA: The Williams Institute. Retrieved from: http://williamsinstitute.law.ucla.edu/wp-content/uploads/geniuss-report-sep-2014.pdf
Gender identity	Q28	Part two of two-step approach on gender identity. The GenIUSS Group (2014) – see Q27 for full reference. Answer options slightly modified.
Sexual orientation identity	Q29	Modified by Generations Study team from SMART (2009). Best practices for asking questions about sexual orientation on surveys. Los Angeles, CA : The Williams Institute. Retrieved from: http://williamsinstitute.law.ucla.edu/wp-content/uploads/SMART-FINAL-Nov-2009.pdf
Sexual behavior	Q30	Modified from SMART report (2009)—see Q29 for full reference
Sexual attraction	Q31	Modified from Reisner, S.L., White Hughto, J.M., Pardee, D., & Sevelius, J. (2015). Syndemics and gender affirmation: HIV sexual risk in female-to-male trans masculine adults reporting sexual contact with cisgender males. *International Journal of STD & AIDS.* Retrieved from: http://www.ncbi.nlm.nih.gov/pubmed/26384946
Multi-group Ethnic Identity Measure-Revised	Q21-Q26	Phinney, J.S. & Ong, A.D. (2007). Conceptualization and measurement of ethnic identity: Current status and future directions. *Journal of Counseling Psychology, 54*(3). Retrieved from: http://isites.harvard.edu/fs/docs/icb.topic1063339.files/Phinney.Ong.2007.pdf
Relationship status	Q32	Meyer, I.H., Dohrenwend, B.P. Schwartz, S. Hunter, J., Kertzner, R.M. (2007). Project Stride Questionnaire. Retrieved from: http://www.columbia.edu/~im15/method/interview.pdf
	Q33-Q35	Modified from Frost, D.M. & Forrester, C. (2013). Closeness discrepancies in romantic relationships: Implications for relational well-being, stability, and mental health. *Personality and Social Psychology Bulletin*, XX(X). Retrieved from:

Construct	Question # in Baseline	Source
		http://m.psp.sagepub.com/content/early/2013/02/13/0146167213476896.full.pdf
	Q36	Created by Generations Study team
Gender conformity and expression	Q37-Q38	Wylie, S.A., Corliss, H.L., Boulanger, V., Prokop, L.A., & Austin, S.B. (2010). Socially assigned gender nonconformity: a brief measure for use in surveillance and investigation of health disparities. *Sex Roles, 63*(3-4).
Sexual/gender labels	Q39	Created by Generations Study team
Identity Centrality subscale	Q40-Q44	Mohr, J.J. & Kendra, M.S. (2012). The Lesbian, Gay, & Bisexual Identity Scale (LGBIS). Measurement instrument database for the Social Science. Retrieved from: http://www.midss.org/sites/default/files/lgbis.pdf
Coming out milestones	Q45-Q51	Modified from Martin JL, & Dean L (1987). Summary of measures: Mental health effects of Aids on at-risk homosexual men. Reference type: Unpublished work
	Q52	Created by Generations Study team
Community connectedness	Q53-Q59	Frost, D.M. & Meyer, I.H. (2011). Measuring community connectedness among diverse sexual minority populations. Journal of Sex Research, 49(1). 36-49. Retrieved from: http://dx.doi.org/10.1080/00224499.2011.565427<br>The Generations Study team used a 7 items instead of the 8 items listed in Frost & Meyer (2011). The last item was not included in the Generations Study because the team was not able to personalize the items to gender and sexual orientation in a way that was useful.
**Healthcare Access & Utilization**		
Healthcare stereotype threat	Q60-Q63	Modified from Abdou, C.M. & Fingerhut, A.W. (2014). Stereotype threat among black and white women in health care settings. *Cultural Diversity & Ethnic Minority Psychology. 20*(3).
Health insurance	Q64	Modified from American Community Survey. Retrieved from: http://www2.census.gov/programs-surveys/acs/methodology/questionnaires/2016/quest16.pdf<br>Modified from U.S. Trans Survey (2015). Unpublished.
Health care utilization	Q65-Q66	National Health Interview Survey (NIHS) (2015).
LGBT specific health	Q68-Q69	Created by Generations Study team based on Generations Study qualitative questions
HIV/STI	Q78-Q79	Composite question modified by Generations Study team based on various surveys about HIV/STI testing
	Q80	Modified and simplified from: Sales, J. M., Spitalnick, J., Milhausen, R. R., Wingood, G. M., DiClemente, R. J., Salazar, L. F., & Crosby, R. A. (2009). Validation of the worry about sexual outcomes scale for use in STI/HIV prevention interventions for adolescent females. *Health Education Research*, *24*(1), 140–152. doi:10.1093/her/cyn006)
	Q81	Composite question created by Generations Study team based on various surveys about HIV testing
PrEP/Truvada	Q82-Q84	Composite question modified by Generations Study team based on various surveys about PReP awareness, attitude, and use
**Health Outcomes**		
Health Related Quality of Life	Q70-Q73	Center for Disease Control and Prevention(CDC) – Behavioral Risk Factor Surveillance System (BRFSS) Survey (2014).

Construct	Question # in Baseline	Source
Physical Health Outcome	Q74	Modified into from NHIS (2014) Adult Survey- Health Outcomes section. Generations Study team created a single check list based on NHIS (2014).
Disability	Q75-Q76	CDC- BRFSS Survey (2014)
Kessler-6	Q77	National Comorbidity Survey. Kessler 6 - Self Report Q1 (a)-(f). Retrieved from: http://www.integration.samhsa.gov/images/res/K6%20Questions.pdf
Alcohol Use	Q85-Q87	Alcohol Use Disorder Identification Test (AUDIT-C)<br>Retrieved from:<br>http://www.integration.samhsa.gov/images/res/tool_auditc.pdf<br>Generations Study team made a slight modification by adding answer option 0.NONE because original AUDIT-C has no skip pattern which could cause confusion to respondents
Tobacco Use	Q88-Q89	CDC- BRFSS Survey (2014)
DUDIT	Q90-Q100	Berman, A.H., Bergman, H., Palmstierna, T., & Schlyter, F. (2003). The Drug Use Disorders Identification Test (DUDIT) Manual.<br>Retrieved from:<br>http://www.paihdelinkki.fi/sites/default/files/duditmanual.pdf
Suicide Behavior	Q101-Q122	Modified from Army – Study to Assess Risk and Resilience in Service Members (STARRS) Instrument. Retrieved from: http://starrs-ls.org/sites/default/files/2016-03/army_starrs_aas_instrument.pdf
**Stressors**		
Concealed Sexual Identity ("Out")	Q123	Meyer, I.H., Rossano, L., Ellis, J.M., Bradford, J.(2002). A brief telephone interview to identify lesbian and bisexual women in random digit dialing sampling. *Journal of Sex Research, 39*. 139-144. One item, degree of being out to "gay, lesbian, or bisexual friends" was not included in the Generations Study.
	Q124	Created by Generations Study team
Felt Stigma	Q125-Q127	Herek (2008), Hate Crimes and Stigma-Related Experiences Among Sexual Minority Adults in the United States. *Journal of Interpersonal Violence.* Retrieved from: http://jiv.sagepub.com/content/early/2008/04/07/0886260508316477.full.pdf+html
Internalized Homophobia-Revised	Q128-Q132	Herek et al (2009), Internalized stigma among sexual minority adults: Insights from a social psychological perspective. *Journal of Counseling Psychology, 56*(1).<br>DOI: 10.1037/a0014672
Conversion treatment	Q133-Q134	Created by Generation Study team based on U.S. Trans Survey (2015). Unpublished.
Victimization and Discrimination	Q135	Herek (2009), Hate Crimes and Stigma-Related Experiences Among Sexual Minority Adults in the United States. *Journal of Interpersonal Violence, 24*(1).
	Q136	Created by Generations Study team based on 1) Krieger N, Sidney S. (1997). Prevalence and health implication of anti-gay discrimination: A study of Black and White women and men in the CARDIA cohort. *International Journal of Health Services.27*:157–176 and<br>2) Williams, D.R., Yu, Y., Jackson, J.S. & Anderson, N.B (1997). Racial differences in physical and mental health: Socioeconomic status, stress,

Construct	Question # in Baseline	Source
		and discrimination. *Journal of Health Psychology, 2*(3).
	Q137-Q138	Modified from 1) Police Public Contact Survey (2011) and 2) Herek (2009)- See Q135 for full reference
	Q139	See Q136 for full reference
	Q140	Modified from 1) Police Public Contact Survey (2011) and 2) Herek (2009)- See Q135 for full reference
	Q141	See Q136 for full reference
Stressful Life Events and Perceived Stress	Q142	National Epidemiologic Survey on Alcohol and Related Conditions (NESARC). (2007)- Wave 2
	Q143	See Q136 for full reference
Everyday Discrimination	Q144	Modified from Williams, D.R., Yu, Y., Jackson, J.S. & Anderson, N.B (1997). Racial differences in physical and mental health: Socioeconomic status, stress, and discrimination. *Journal of Health Psychology, 2*(3).
	Q145	See Q136 for full reference
Chronic Strains	Q146	Abridged version from Wheaton B. The nature of stressors. In: Horwitz AF, Scheid TL, editors. *A handbook for the study of mental health: Social contexts, theories, and systems*. Cambridge, UK: Cambridge University Press; 1999. pp. 176–197.
Childhood gender conformity	Q147-Q150	Selected measure from Zucker, K.J., Mitchell, J.N., Bradley, S.J., Tkachuk, J. Cantor, J.M. & Allin, S.M.(2006), The Recalled Childhood Gender Identity/Gender Role Questionnaire: Psychometric properties. *Sex Roles, 54*(7).
Adverse Childhood Experiences	Q151-Q161	CDC-BRFSS (2010). Adverse Childhood Experiences (ACE) module. Retrieved from: http://www.acestudy.org/
Bullying	Q162	Composite question created by Generations Study team based on various surveys about childhood bullying
	Q163	See Q136 for full reference
Neighborhood acceptance	Q19	Answer options modified from Gallup World Poll (2008) survey question
**Social Support**		
Multidimensional scale of perceived social support	Q164	Zimet, G.D., Dahlem, N.W., Zimet, S.G. & Farley, G.K. (1988). The Multidimensional Scale of Perceived Social Support. *Journal of Personality Assessment, 52*, 30-41. Retrieved from: http://www.yorku.ca/rokada/psyctest/socsupp.pdf
**Demographics**		
Year of birth	Q165	National Survey of Drug Use and Health (2014)
Nativity	Q166-Q168	Modified from National Survey of Drug Use and Health (2014)
Race/Ethnicity	Q20	Created by Generations Study team based on surveys asking about race/ethnicity
Children	Q169-Q170	Modified from U.S. Trans Survey (2015) unpublished and CDC-BRFSS 2014
Employment	Q171	Gallup Survey
Income	Q172-Q174	Gallup Survey
Wealth	Q175	Project Stride Questionnaire (2007) – See Q32 for full reference. Item adapted from Conger, R.D., Wallace, L.E., Sun, Y., Simmons, R.L., McLoyd, V.C., Brody, G.H. (2002). Economic pressure in African American families: A replication and extension of the family stress model.

Construct	Question # in Baseline	Source
		Developmental Psychology, 38, 179-193.
Home ownership	Q176	CDC-BRSFF (2014)
Housing stability	Q177-Q178	Modified from M. Vijayaraghavan, M.B. Kushel, E. Vittinghoff, et al. (2013). Housing Instability and Incident Hypertension in the CARDIA Cohort. *Journal of Urban Health, 90*(3) 427-441
Religiosity	Q179-Q181	Modified from Pew Research Center (2013)- A survey of LGBT Americans
Military service experience	Q182-Q1	Created by Generations Survey team
